# Comparative Analysis of Endovascular Intervention and Endarterectomy in Patients with Femoral Artery Disease: A Systematic Review and Meta-Analysis

**DOI:** 10.3390/hematolrep14020026

**Published:** 2022-06-01

**Authors:** Nidhruv Ravikumar, Gopika Sreejith, Sharon Hiu Ching Law, Prakhar Anand, Noah Varghese, Samrin Kagdi, Navneet Kang, Mohamed Nashnoush, Sihat Salam, Ibsen Ongidi

**Affiliations:** 1 Department of Medicine, School of Medicine, Queen’s University Belfast, Belfast BT7 1NN, UK; gsreejith01@qub.ac.uk; 2 RadScholars Inc., Halifax, NS B3H 4R2, Canada; 20shl1@michener.ca (S.H.C.L.); prakhar.anand@mail.utoronto.ca (P.A.); nvarghe@uwo.ca (N.V.); samrinekagdi@gmail.com (S.K.); kangn11@mcmaster.ca (N.K.); mnashnoush@dal.ca (M.N.); sihat.s00@gmail.com (S.S.); ibsenongidi@students.uonbi.ac.ke (I.O.); 3 Department of Radiation Oncology, University of Toronto, Toronto, ON M5S 1A1, Canada; 4 Department of Medicine, National University of Ireland, Galway, H91 TK33 Galway, Ireland; 5 Department of Biochemistry, Western University, London, ON N6A 3K7, Canada; 6 Department of Biological Sciences and Health and Society, University of Toronto, Toronto, ON M5S 1A1, Canada; 7 Department of Chemistry and Chemical Biology, McMaster University, Hamilton, ON L8S 4L8, Canada; 8 School of Health Sciences, Dalhousie University, Halifax, NS B3H 4R2, Canada; 9 IWK Health Center, Halifax, NS B3K 6R8, Canada; 10 Department of Biomedical science, York University, Toronto, ON M3J 1P3, Canada; 11 Department of Human anatomy, School of Medicine, University of Nairobi, Nairobi P.O. Box 30197-00100, Kenya

**Keywords:** endovascular, endarterectomy, femoral artery, atherosclerosis, minimally invasive, peripheral arterial disease, stent

## Abstract

Peripheral artery disease is a prevalent illness affecting more than 200 million people worldwide. A commonly used technique to manage the condition has been open endarterectomy. However, in recent times, a shift towards minimally invasive techniques has resulted in endovascular intervention as a popular alternative. This review aims to assess the safety and efficacy of endovascular intervention when compared with endarterectomy. A systematic review of the articles published in PubMed, Ovid, Embase, and Scopus within the last 10 years was conducted. The PRISMA guidelines were adhered to, and the Newcastle-Ottawa and NICE quality assessment scales were used. A meta-analysis of proportions was performed using the RStudio software (RStudio Team (2021). RStudio: Integrated Development Environment for R, PBC, Boston, MA, USA). Twenty-six studies were included, with a total of 7126 patients (endovascular, 2496; endarterectomy, 4630). Technical success was greater for endarterectomy than endovascular intervention with an odds ratio of 0.38; 95% CI [0.27–0.54]. In terms of safety as well endovascular intervention was better than endarterectomy with an odds ratio of 0.22; 95% CI [0.15 to 0.31] for wound infection. Endovascular intervention is a safe and effective procedure; however, it cannot be considered superior to endarterectomy.

## 1. Introduction

Peripheral artery disease (PAD) is an umbrella term that describes the circulatory compromise in the lower limb arteries due to atherosclerotic plaque build-up. When this involves the femoral arteries, PAD is known as femoral artery disease (FAD). PAD is prevalent among 10% of the American population [1], predominantly in patients over 40 years of age. Up to 50% of individuals show visible symptoms [2]. The most common presenting symptom patients experience is claudication. This refers to a vague discomfort, pain, or fatigue that occurs because of ischaemic changes upon exercise. In FAD, the thigh and the upper two-thirds of the calf are often affected [1]. The primary aim of managing the condition is to modify the risk factors and decrease overall cardiovascular risk. These include lifestyle changes, such as exercise, smoking cessation, and diet modification, along with the use of medications, such as antihypertensives and lipid-lowering drugs. However, in many cases, there still exists some level of disability despite aggressive medical therapy and lifestyle modifications. In such cases, the need for revascularisation is paramount. Various techniques are employed to achieve this, including endarterectomy and an endovascular approach [1]. Femoral artery endarterectomy (FAE) is an open procedure that is performed through an incision in the patient’s groin region, resecting the embedded plaque in the femoral artery. The procedure is known to have a high technical success rate and low mortality. However, it is associated with significant local morbidity, such as wound infections, wound haematomas, and other wound-related complications, affecting more than 15% of patients [3]. In recent years, a shift has been seen from FAE to endovascular treatment (ET) owing to continuous advances in the field. This is a minimally invasive procedure that involves the placement of a stent within the artery that helps in establishing blood flow [4]. This method has further been developed into FDA-approved drug-eluting stents that consist of biodegradable polymers that dissolve into the patient’s bloodstream over time after clearing the occlusion. Because of its minimally invasive nature, it is associated with a shorter length of hospital stay and decreased wound-related complications [5]. This systematic review aims to compare and contrast the efficacy and safety of FAE and ET.

## 2. Materials and Methods

### 2.1. Information Source and Search

An electronic literature search was executed on PubMed, Ovid, Embase, and Scopus to obtain relevant articles to perform a comprehensive comparative analysis of FAE and ET. Following the Preferred Reporting Items for Systematic Reviews and Meta-analysis (PRISMA) guidelines, articles relevant to the research topic were identified. No authors were contacted for additional studies. Keywords used were: “Femoral artery”, “Stents”, “Endovascular”, and “Endarterectomy” in combination with MeSH terms. An example of a search strategy used for PubMed was as follows: “endarterectomy*” (MeSH) OR “endovascular procedure*” (MeSH) OR “stent*” (MeSH) OR “endarterectomy*” (tw) OR “endovascular” (tw) OR “stent*” (tw) AND “femoral artery*” (MeSH) OR “peripheral arterial disease*” (MeSH) OR “femoral artery*” (tw). The search strategy employed for other databases is mentioned in Supplementary File S1. The initial search yielded 6192 publications in PubMed, 740 publications in Embase, and 1607 publications in Scopus. These were further refined to ensure that the inclusion and exclusion requirements were met. Relevant articles from each search engine were extracted, and duplicates were omitted such that 26 articles remained, from which data were extracted.

### 2.2. Eligibility Criteria

We included primary research articles published in English within the last 10 years (2011–2021) because of the recent advancements in minimally invasive techniques within the past decade. The study subjects were limited to humans only, and there was no restriction on the length of follow-up. This research aimed at collecting data from papers discussing the endovascular and endarterectomy intervention in FAD. Papers reporting interventions on any other lower limb arteries were excluded. Case series (retrospective and prospective studies) and clinical trials were considered acceptable, with a majority of the studies selected being primarily retrospective analysis studies, prospective studies, and sub-analysis studies. Studies in which the subjects were treated for graft stenosis, thrombangiitis, arteritis, or trauma were excluded, as they are not representative of the population under study (femoral artery atherosclerosis). Further criteria for exclusion consisted of studies in which patients underwent concomitant bypass surgery. 

### 2.3. Outcomes Assessed

Efficacy and safety outcomes were identified for FAE and ET and extracted from the included studies.

A division of the outcomes assessed into primary and secondary on the basis of direct correlation to the parameters assessed was made. The efficacy of the procedures was primarily assessed by the technical success reported. Technical success was defined as residual stenosis of <30% on peri-operative angiography. For other outcomes, such as symptom improvement, the requirement for further revascularisation and failure to salvage limbs resulting in amputation were considered secondary outcomes assessing efficacy.

Similarly, short- and long-term mortality and wound infection were considered the primary outcomes to assess safety. The incidence of myocardial infarction, wound haematoma, dehiscence, stent fractures, other perioperative complications, and the requirement for further surgery to treat adverse effects were the secondary outcomes assessed.

The long-term and short-term effects were also analysed for the two treatment procedures. Long-term outcomes included technical success, stent fracture at follow-up, mortality, and delayed wound healing. Short-term outcomes were categorised into peri-operative complications; wound-related complications, which included wound haematoma and wound infection; myocardial infarction; amputation; and further surgery for adverse events. 

### 2.4. Study Selection and Data Collection

Study selection was based on meeting the inclusion and exclusion criteria stated previously. Three reviewers were involved in the initial search and screened the article abstracts. Following this, the full article was assessed, ensuring relevance to FAE and endovascular. Furthermore, three additional reviewers evaluated the quality of the studies selected to confirm that the studies were apt for data extraction. In cases in which the full text was not available, study researchers were not contacted.

### 2.5. Risk of Bias in Individual Studies 

Following the PRISMA guidelines, articles relevant to the topic were identified. The PRISMA checklist was used to omit bias. The Newcastle–Ottawa quality assessment scale was used to assess the quality of observational studies and case–control studies. To assess the quality of RCTs, another checklist was used that was developed by the Review Body for Interventional Procedures (Health Services Research Units at the Universities of Aberdeen and Sheffield), which is an independent review body that conducts systematic reviews for the Interventional Procedures Programme of the UK National Institute for Health and Care Excellence (NICE). The respective checklists are included in Supplementary Files S2 and S3. The aforementioned checklists ensured transparency in the study selection process. Additional measures were taken to make sure a holistic analysis of the selected studies was screened more than once for quality control.

### 2.6. Summary Measure and Synthesis of Results 

The factors assessed in the comparative analysis between ET and FAE were tabulated. The technical success criteria considered included the success rate of the procedure, conversion to surgery, improvement of symptoms, need for revascularisation, and failure of limb salvage. Furthermore, the safety outcomes measured were as follows: short- and long-term mortality, the incidence of myocardial infarction, wound infection, wound haematoma, dehiscence, other wound complications, stent fractures, other perioperative complications, and further surgery for adverse events. The event rate, odds ratio, and confidence intervals were reported for each parameter. Calculations of all relevant statistical variables were conducted using RStudio software (RStudio Team (2021). RStudio: Integrated Development Environment for R, PBC, Boston, MA, USA).

### 2.7. Statistical Analysis 

The primary statistical variable was calculated as the event rate for each parameter assessed under technical success and safety outcomes. A meta-analysis of proportions was then performed to determine the pooled effect size for each parameter. Using the pooled estimate for each of the two procedures, the odds ratio was calculated. The odds ratio was calculated as the ratio between endovascular procedures and endarterectomy such that endarterectomy was used as a control. The sorting process of the ratio was as follows: if it was greater than 1, the likelihood of the event occurring in the ET group was higher than the likelihood of it occurring in the FAE group. Consequently, an odds ratio of less than 1 signified the likelihood of the event occurring in the FAE group was higher than the likelihood of it occurring in the ET group. Finally, if the odds ratio included a 1, then it was considered nonsignificant. An alpha/significance (*p*-value) level of 0.05 was used in the assessment of whether the results obtained were significant at a 95% confidence interval. A *p*-value of less than or equal to 0.05 suggested the results were statistically significant.

## 3. Results

### 3.1. Study Characteristics

**Figure 1:**
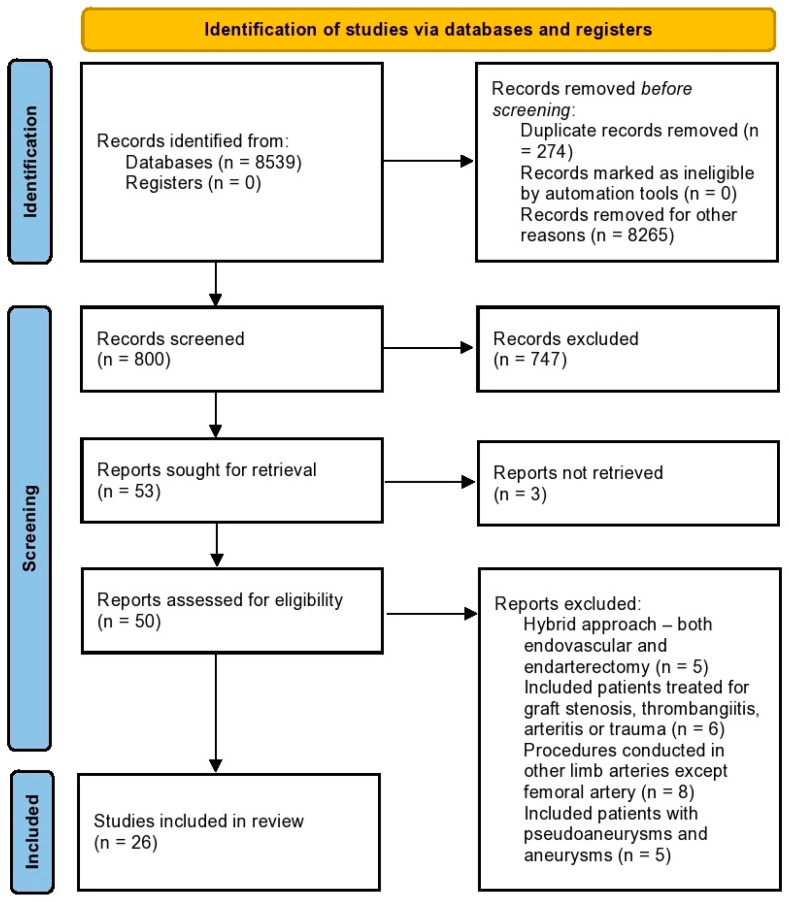
PRISMA flowchart (other reasons—review articles, studies describing the treatment of carotid/iliac/coronary arteries, and the use of hybrid techniques).

Initially, 8539 potential studies were found and examined, with 274 duplicates. Fifty-three articles were sought for retrieval after screening the titles and abstracts. From these reports, 26 articles published between the years 2011 and 2021 were included after screening the full article for eligibility (see Figure 1). A total of 7126 patients (endovascular, 2496; endarterectomy, 4630) were included in these studies. Two of the studies were RCTs, twenty-one were retrospective studies, and three were prospective studies. The retrospective and prospective cohort studies were grouped as case series for this study. The mean follow-up period was 2.96 years (range: 1 month–12.5 years). These data are summarised in Table 1.

### 3.2. Risk of Bias

Data regarding the quality of the studies included are tabulated in the Supplementary Files Table S1 for RCTs and Table S2 for case series. A total of 2 RCTs were included as part of this systematic review. The first RCT [6] was of particularly high quality. The study included 80 patients from a single centre who underwent random allocation to the FAE or ET group. The patients were followed up for 9 months in the endovascular group and 11 months in the endarterectomy group. The primary outcome measure was surgical site infections. This study was considered to be of high quality because of effective treatment concealment along with the conduction of an intention-to-treat analysis. The second RCT [7], however, was of lower quality since no direct comparison was made between FAE and ET. The study included 95 patients randomly allocated to either the remote endarterectomy or ET groups. Of the 95 patients included, 44 underwent endovascular intervention and were considered in our study. Although the study was considered to be of low quality, there was effective blinding, and all patients were treated equally. 

Twenty-four cohort studies were included as part of this study. There were a total of 3 prospective studies and 21 retrospective analyses. Because the majority of studies included were retrospective, there is a risk of selection bias. However, all the papers included were considered to be of high quality when analysed using the Newcastle–Ottawa Scale. In terms of patient selection and definition of the controls, all studies were considered to be of high quality. There were a few studies that did not include an adequate follow-up period and hence may have resulted in bias. 

Overall, the quality of studies included was of a high standard; however, there may be a possibility of bias.

### 3.3. Patient Characteristics

A total of 7126 patients were considered in the 26 studies, with 4861 males and 2689 females. The endovascular intervention reports included a total of 2496 patients (1763 males and 978 females). On the other hand, the endarterectomy reports included 4630 patients (2098 males and 1711 females). The two RCTs had a total of 164 patients, with the rest belonging to retrospective and prospective studies. The average age of participants in all 26 studies was 68.70 years old, with an average age of 68.24 for endovascular intervention and an average of age 69.16 years for endarterectomy. 

### 3.4. Technical Success 

**Figure 2:**
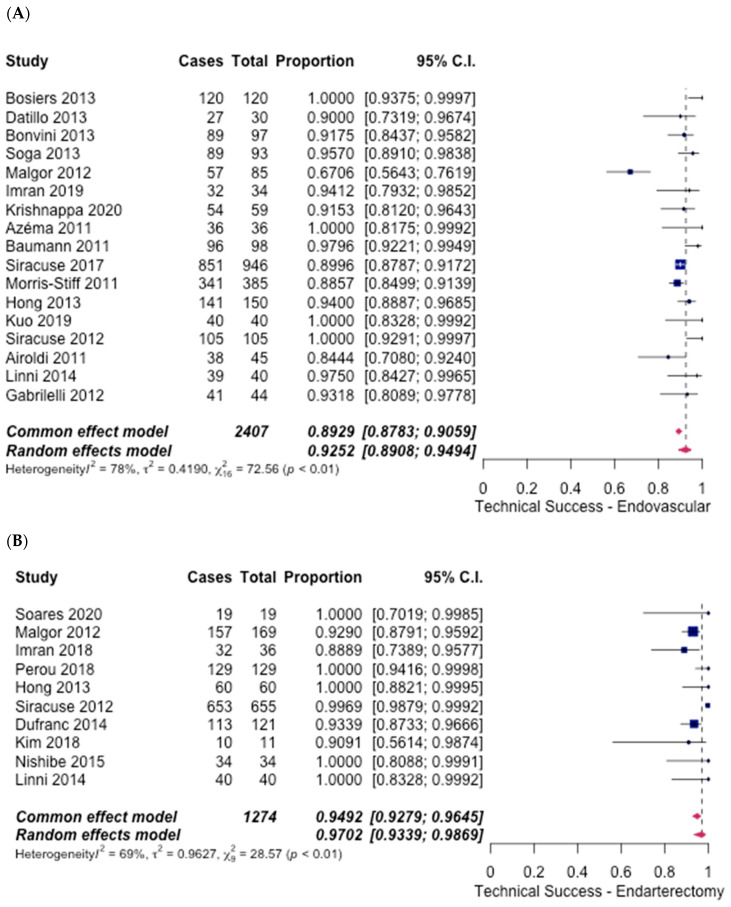
Forest plot for technical success. (**A**) Endovascular intervention [6,7,8,9,10,11,12,13,14,15,16,17,18,19,20,21,22]. (**B**) Endarterectomy [5,6,13,14,20,22,23,24,25,26].

A primary outcome that was looked at was technical success. High technical success rates were observed in both groups. In the ET group, 17 studies [6,7,8,9,10,11,12,13,14,15,16,17,18,19,20,21,22] reported technical success. An overall success rate of 92.52% (95% CI [89.08%, 94.94%]) was noted (Figure 2A). However, in the FAE group, 10 studies [6,7,13,14,20,22,23,24,25,26] reported the technical success rates, and an overall success rate of 97.02% (95% CI [93.39%, 98.69%]) (Figure 2B) was observed. The odds ratio calculated was 0.38 (95% CI [0.27–0.54], *p* < 0.001), thus favouring the FAE group significantly. 

**Figure 3:**
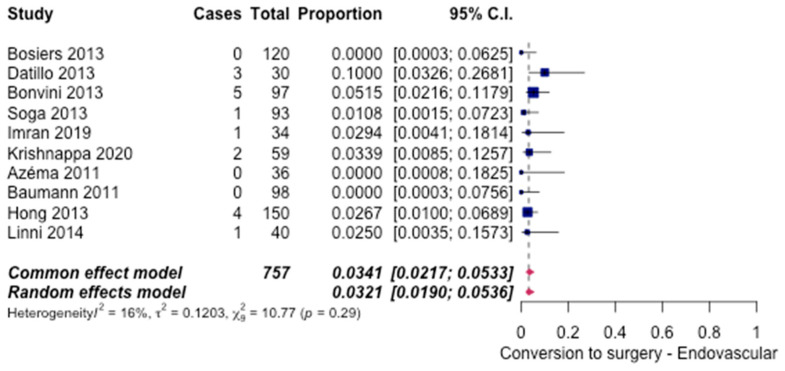
Forest plot for conversion to surgery in endovascular intervention [6,8,10,11,12,14,15,16,17,20].

The subsequent need for surgery was reported in a few cases of ET. 10 studies [6,8,10,11,12,14,15,16,17,20] reported a need for further surgical interventions. Overall, 3.21% (95% CI [1.90%, 5.36%]) (Figure 3) of the cases required conversion to open surgery.

**Figure 4:**
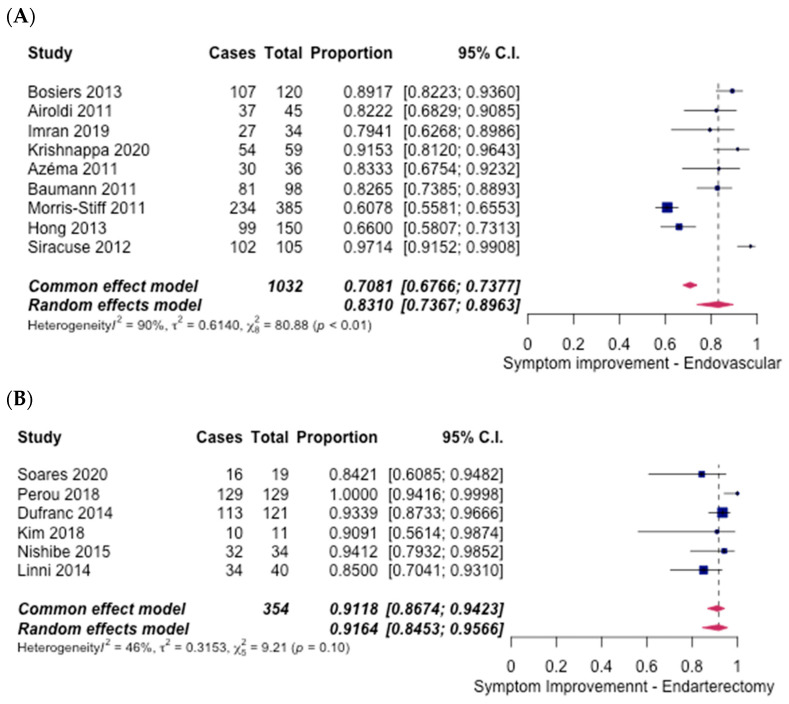
Forest plot for symptom improvement. (**A**) Endovascular intervention [8,9,14,15,16,17,18,19,20]. (**B**) Endarterectomy [5,6,23,24,25,26].

Symptom improvement was reported in 9 case series [8,9,14,15,16,17,18,19,20] for ET procedures, while under FAE treatment, 1 RCT [6] and 5 case series [5,23,24,25,26] commented on symptom improvement. For ET procedures, the overall rate of symptom improvement was 83.10% (95% CI [73.67%, 89.63%]) (Figure 4A). However, for FAE, the overall rate was 91.64% (95% CI [84.53%, 95.66%]) (Figure 4B). An odds ratio of 0.45 (95% CI [0.30–0.67], *p* < 0.05) was calculated, favouring the FAE group.

**Figure 5:**
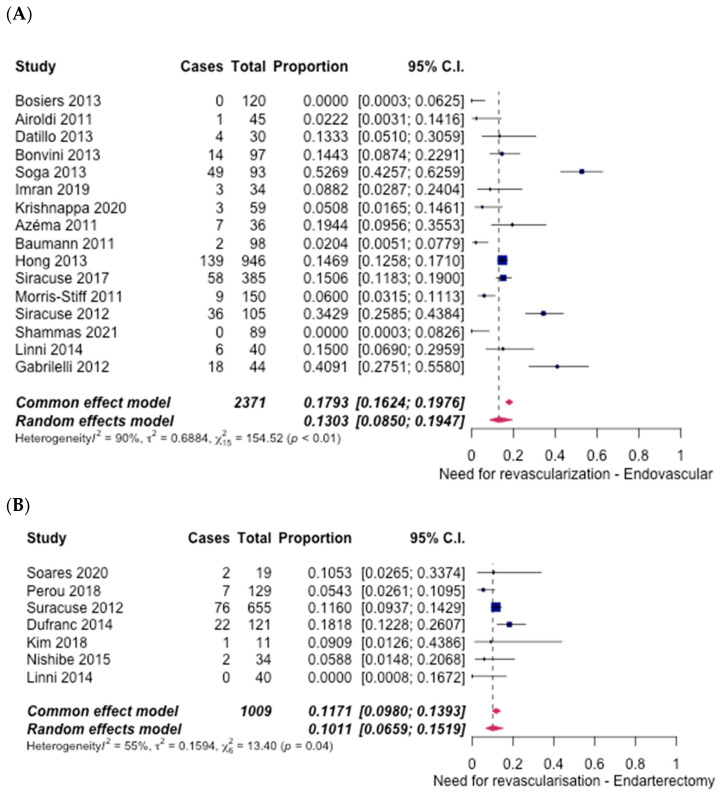
Forest plot for need for revascularisation. (**A**) Endovascular intervention [6,7,8,9,10,11,12,14,15,16,17,18,19,20,22,27]. (**B**) Endarterectomy [5,6,22,23,24,25,26].

The need for revascularisation was considered to be low in both procedures. In the ET group, 16 studies [6,7,8,9,10,11,12,14,15,16,17,18,19,20,22,27] reported this, with an overall rate of 13.03% (95% CI [8.5%, 19.47%]) (Figure 5A). In the case of FAE, seven studies [5,6,22,23,24,25,26] discussed the need for revascularisation, with an overall rate of 10.11% (95% CI [6.59%, 15.19%]) (Figure 5B). An odds ratio of 1.33 (95% CI [1.05–1.69], *p* < 0.05) was calculated, indicating higher rates in the ET group.

**Figure 6:**
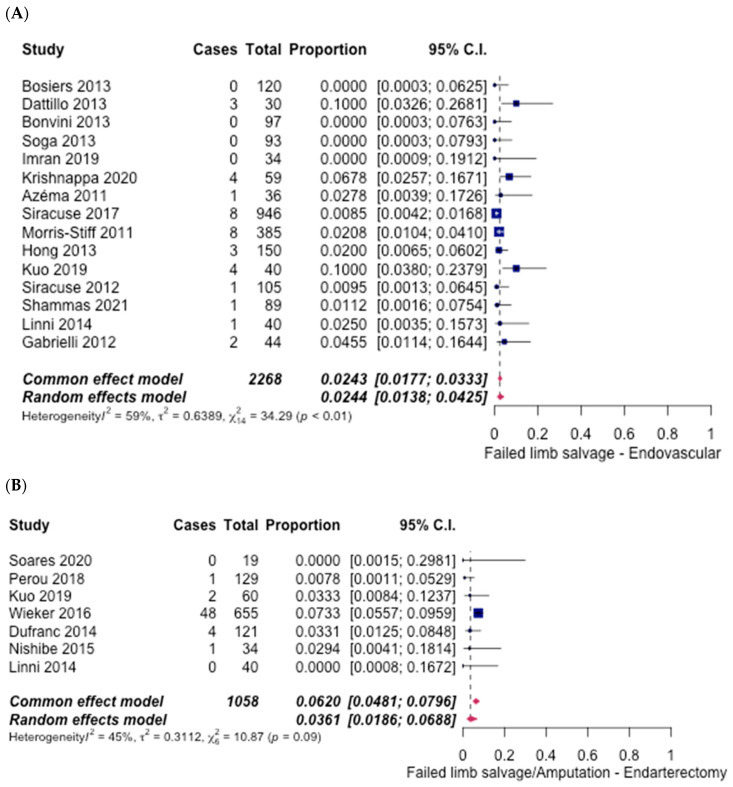
Forest plot for failed limb salvage/amputation. (**A**) Endovascular intervention [6,7,8,10,11,12,14,15,16,18,19,20,21,22,27]. (**B**) Endarterectomy [6,21,23,24,25,26,28].

There was a low incidence of amputation for both treatment options discussed. In the ET group, 15 studies [6,7,8,10,11,12,14,15,16,18,19,20,21,22,27] discussed the incidence of amputation. Amongst these studies, a total of 2.44% (95% CI [1.38%, 4.25%]) (Figure 6A) of the patients underwent amputation. By contrast, in the FAE group, seven studies [6,21,23,24,25,26,28] reported rates of amputation, of which 3.61% (95% CI [1.86%, 6.88%]) (Figure 6B), required an amputation. An odds ratio of 0.67 (95% CI [0.44–1.02], *p* = 0.88) indicated no significant difference between the two groups.

### 3.5. Safety Outcomes 

**Figure 7:**
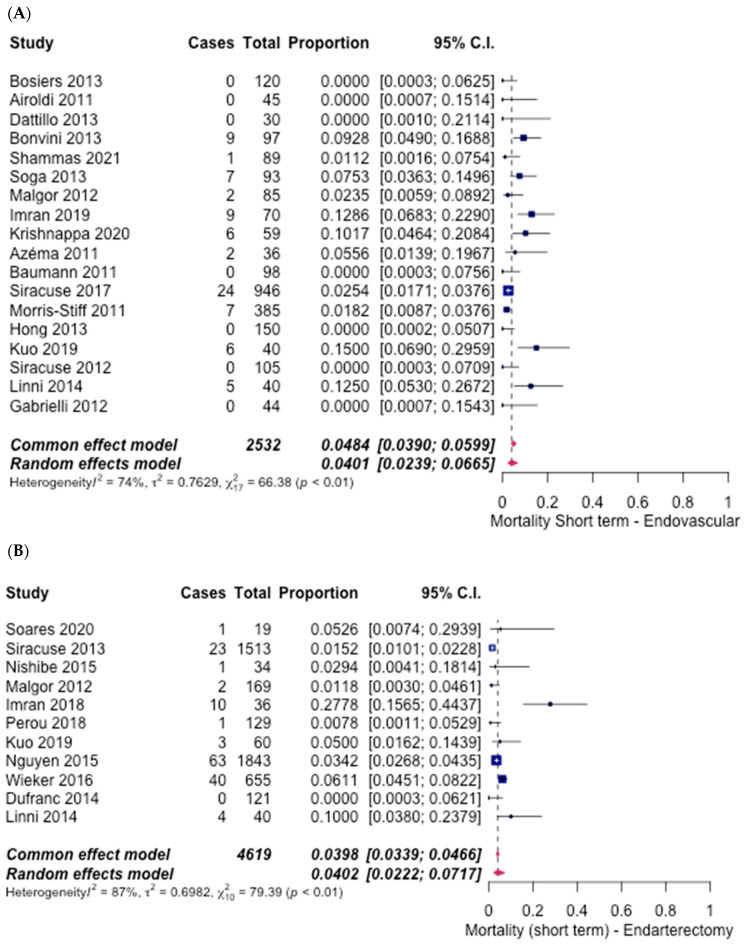
Forest plot for short-term mortality. (**A**) Endovascular intervention [6,7,8,9,10,11,12,13,14,15,16,17,18,19,20,21,22,27]. (**B**) Endarterectomy [6,13,14,21,23,24,25,26,28,29,30].

Analysing the short-term mortality rates, the ET group included 18 studies [6,7,8,9,10,11,12,13,14,15,16,17,18,19,20,21,22,27], with an overall rate of 4.01% (95% CI [2.39%, 6.65%]) (Figure 7A), and the FAE group included 11 studies [6,13,14,21,23,24,25,26,28,29,30], with an overall rate of 4.02% (95% CI [2.22%, 7.17%]) (Figure 7B). The cumulative odds ratio suggested that there were no significant differences observed between the two groups. The odds ratio was 0.99 (95% CI [0.90–1.10], *p* = 1.06). 

**Figure 8:**
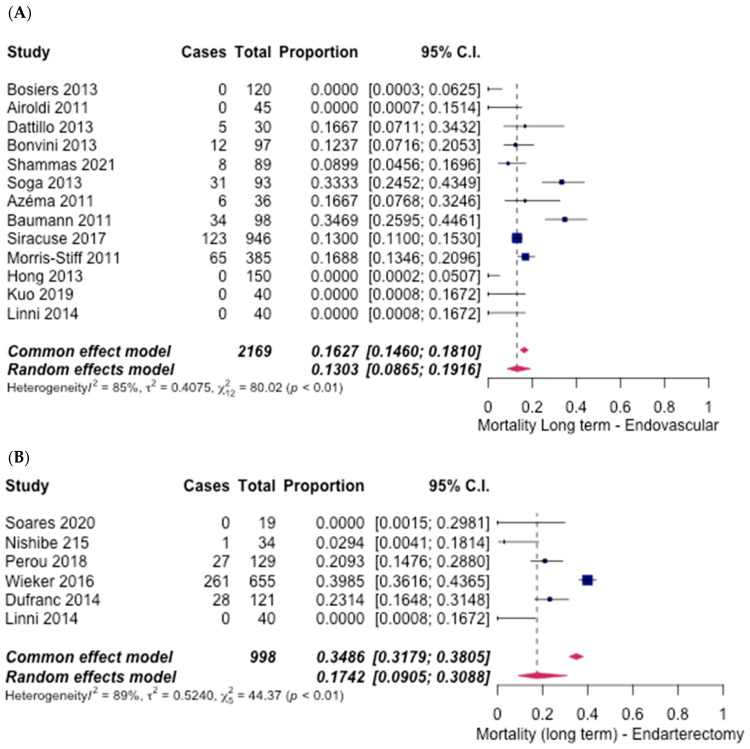
Forest plot for long-term mortality. (**A**) Endovascular intervention [6,8,9,10,11,12,16,17,18,19,20,21,27]. (**B**) Endarterectomy [6,23,24,25,26,28].

Similarly, long-term mortality rates from all studies showed no significant difference between groups, with an odds ratio of 0.71 (95% CI [0.58 to 1.06], *p* = 0.63). The ET group had 13 studies [6,8,9,10,11,12,16,17,18,19,20,21,27], with a total of 13.03% (95% CI [8.65%, 19.16%]) (Figure 8A) experiencing long-term mortality. Six studies [6,23,24,25,26,28] in the FAE group reported an overall rate of 17.42% (95% CI [9.05%, 30.88%]) (Figure 8B). 

**Figure 9:**
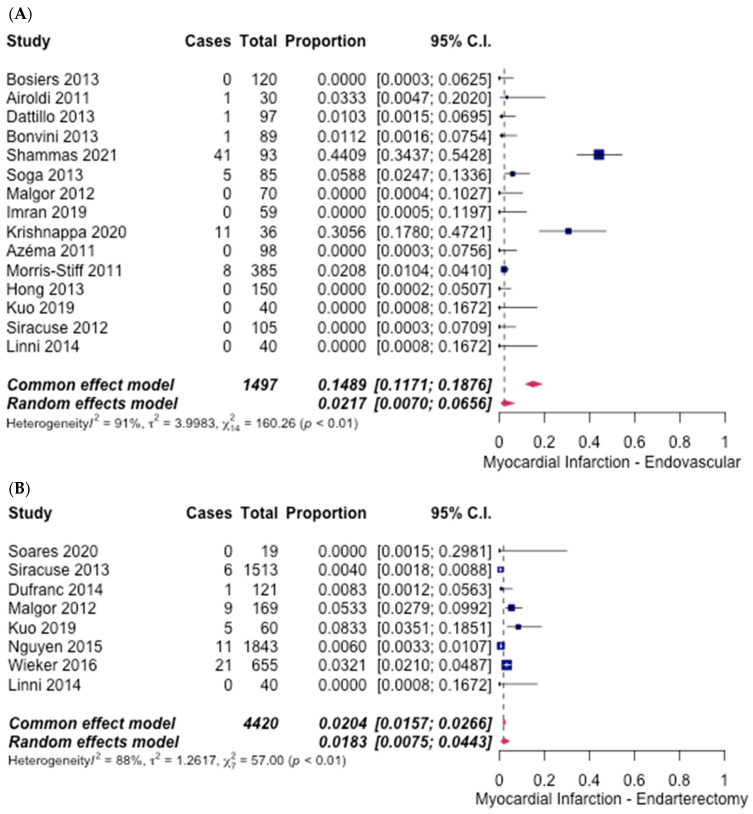
Forest plot for myocardial infarction. (**A**) Endovascular intervention [6,8,9,10,11,12,13,14,15,16,19,20,21]. (**B**) Endarterectomy [6,13,21,23,25,28,29,30].

Myocardial infarction was reported in 15 studies [6,8,9,10,11,12,13,14,15,16,19,20,21] in the ET group, and the incidence rate was 2.17% (95% CI [0.7%, 6.56%]) (Figure 9A). In the FAE group, the incidence of MI was reported in eight studies [6,13,21,23,25,28,29,30], and an overall rate of 1.83% (95% CI [0.75%, 4.43%]) (Figure 9B) was noted. The odds ratio calculated was 1.19 (95% CI [0.79–1.63], *p* = 0.35), indicating no significant difference.

**Figure 10:**
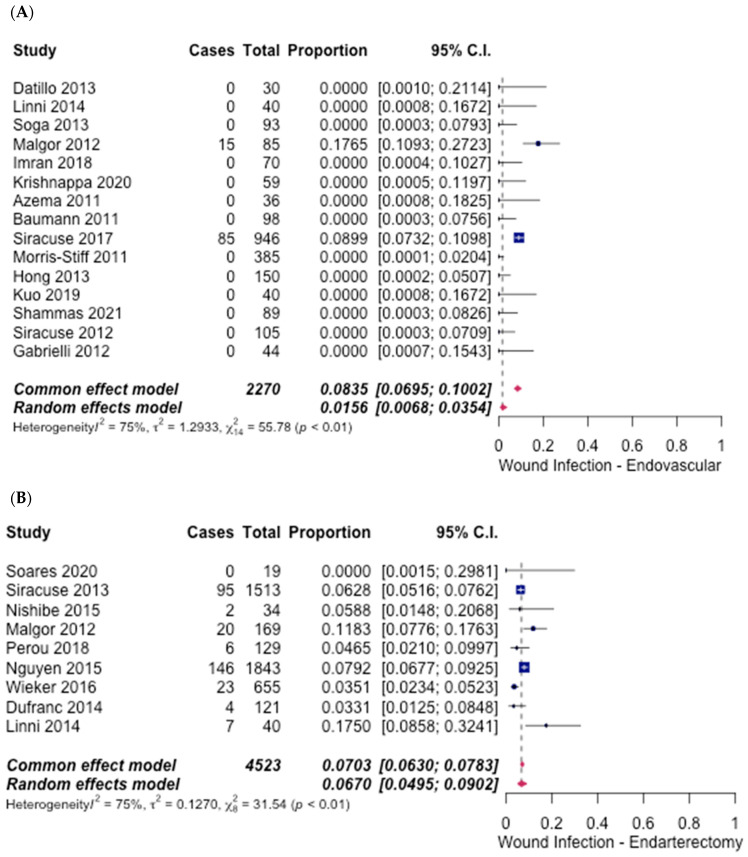
Forest plot for wound infection. (**A**) Endovascular intervention [6,7,10,12,13,14,15,16,17,18,19,20,21,22,27]. (**B**) Endarterectomy [6,23,24,25,26,28,29,30].

Wound infection was reported in 1.56% (95% CI [0.68%, 3.54%]) (Figure 10A) of ET (15 studies) [6,7,10,12,13,14,15,16,17,18,19,20,21,22,27] and 6.70% (95% CI [4.95%, 9.02%]) (Figure 10B) of FAE (nine studies) [6,23,24,25,26,28,29,30]. The odds ratio was 0.22 (95% CI [0.15–0.31], *p* < 0.05), in favour of ET.

**Figure 11:**
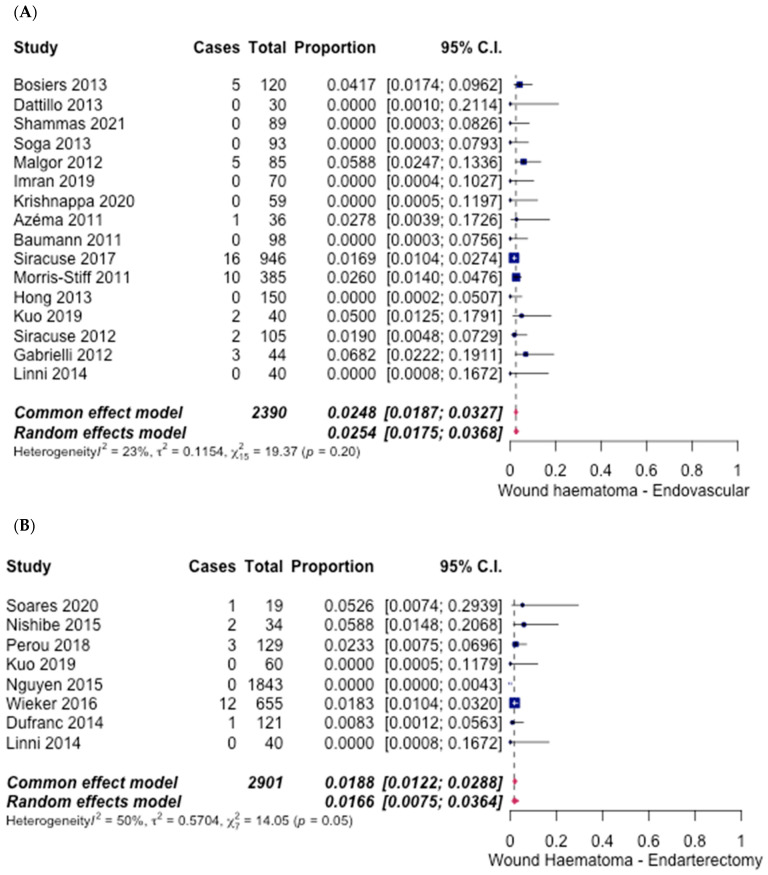
Forest plot for wound haematoma. (**A**) Endovascular intervention [6,7,8,10,12,13,14,15,16,17,18,19,20,21,22,27]. (**B**) Endarterectomy [6,21,23,24,25,26,28,29].

Reporting across the 16 studies [6,7,8,10,12,13,14,15,16,17,18,19,20,21,22,27] on ET and 8 studies [6,21,23,24,25,26,28,29] on FAE showed a significant difference between each group for wound haematoma. The pooled proportion of patients in the ET group experiencing wound haematoma was 2.54% (95% CI [1.75%, 3.68%]) (Figure 11A), and in the FAE group, it was 1.66% (95% CI [0.75%, 3.64%]) (Figure 11B). The odds ratio was 1.54 (95% CI [1.05–2.26], *p* < 0.05).

**Figure 12:**
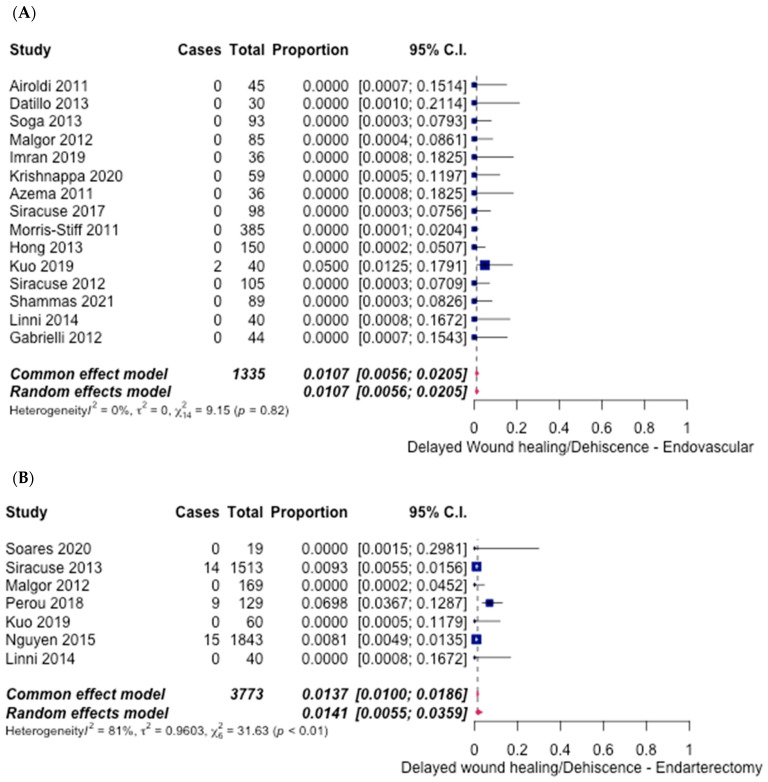
Forest plot for delayed wound healing/dehiscence. (**A**) Endovascular intervention [6,7,9,10,12,13,14,15,16,18,27]. (**B**) Endarterectomy [6,13,21,23,24,29,30].

Delayed wound healing and dehiscence occurred in 1.07% (95% CI 0.56%, 2.05%]) (Figure 12A) of ET procedures (15 studies) [6,7,9,10,12,13,14,15,16,18,27], in contrast to 1.41% (95% CI [0.55%, 3.59%]) (Figure 12B) of FAE (seven studies) [6,13,21,23,24,29,30]. An odds ratio of 0.76 (95% CI [0.42–1.37], *p* = 1.36) was calculated, indicating no significant difference. 

**Figure 13:**
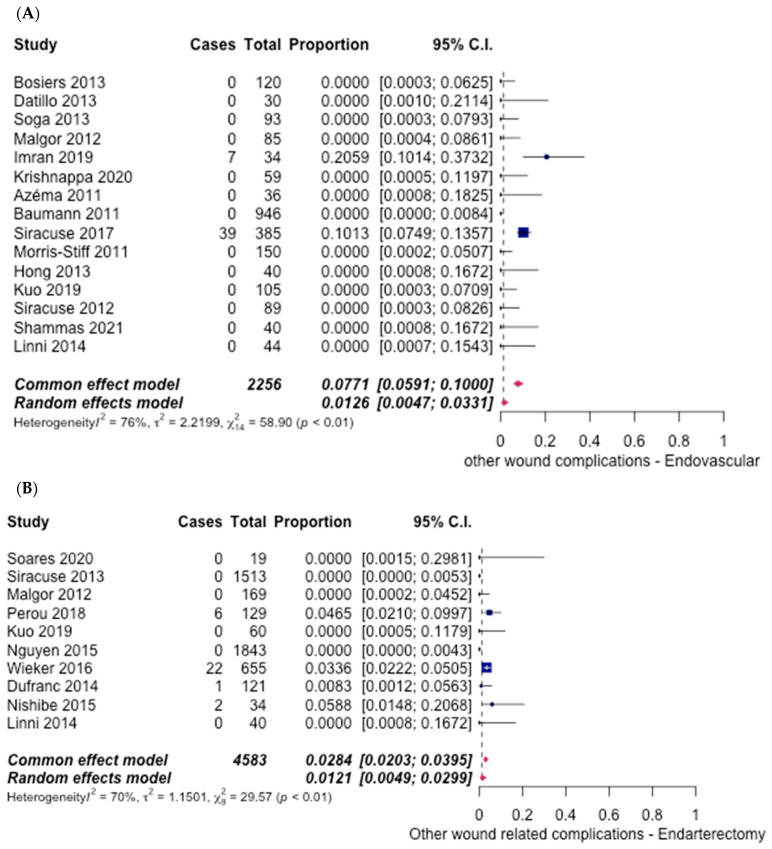
Forest plot for other wound-related complications. (**A**) Endovascular intervention [6,8,10,12,13,14,15,16,17,18,19,20,21,22,27]. (**B**) Endarterectomy [6,13,21,23,24,25,26,28,29,30].

Other wound-related complications occurred in 16 studies [6,8,10,12,13,14,15,16,17,18,19,20,21,22,27] for ET {1.26% (95% CI [0.47%, 3.31%])} (Figure 13A) and 10 studies [6,13,21,23,24,25,26,28,29,30] for FAE {1.21% (95% CI [0.49%, 2.99%])} (Figure 13B). No significant difference was noted between the two groups, with an odds ratio of 1.04 (95 CI [0.67–1.63], *p* = 0.87). 

**Figure 14:**
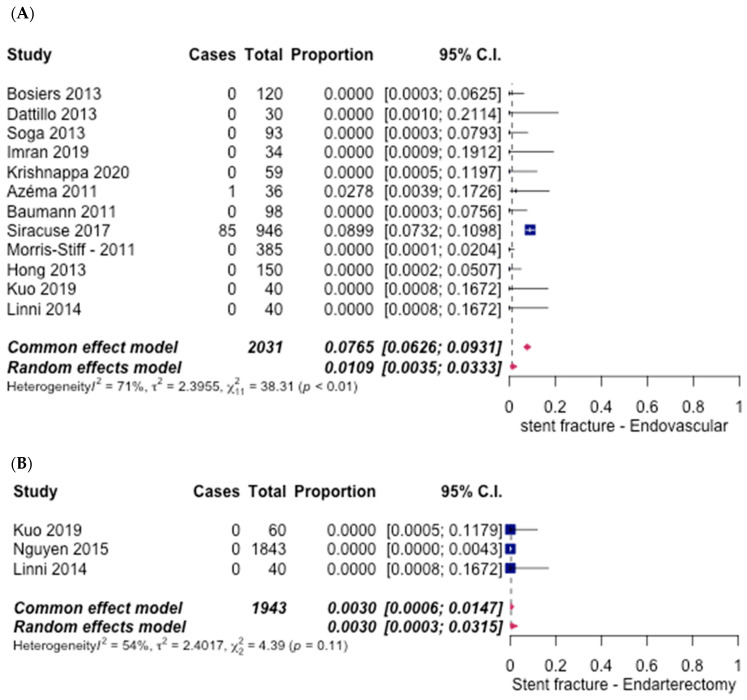
Forest plot for stent fracture. (**A**) Endovascular intervention [6,8,10,12,14,15,16,17,18,19,20,21]. (**B**) Endarterectomy [6,21,29].

The rates of stent fracture were reported in 12 studies [6,8,10,12,14,15,16,17,18,19,20,21] for ET and 3 studies [6,21,29] for FAE. The ET group observed an overall rate of only 1.09% (95% CI [0.47%, 3.33%]) (Figure 14A), while the FAE group reported an overall rate of 0.3% (95% CI [0.03%, 3.15%]) (Figure 14B). The pooled odds ratio was 3.66 (95% CI [1.48–9.05], *p* < 0.05), in favour of the FAE group.

**Figure 15:**
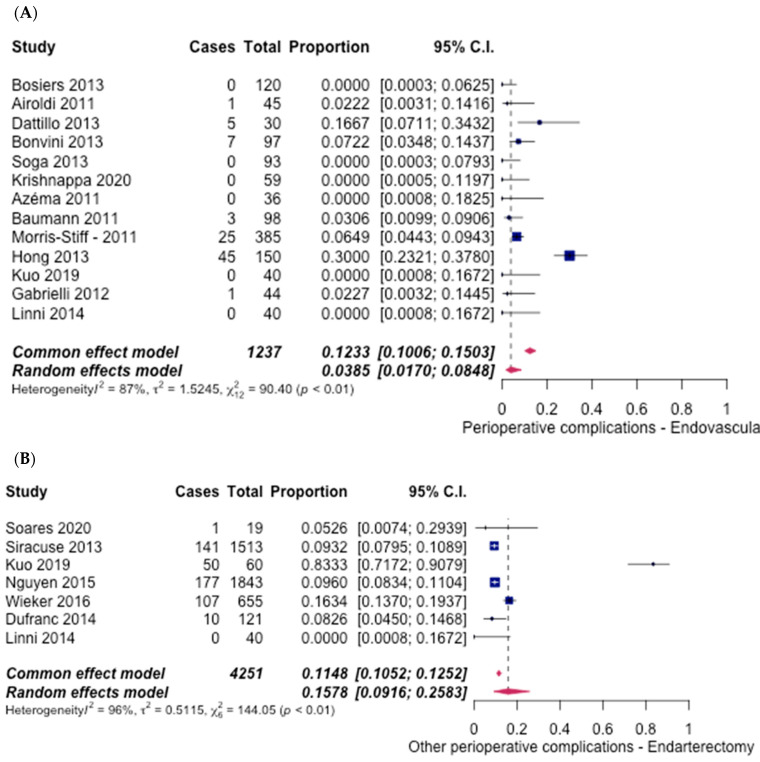
Forest plot for other perioperative complications. (**A**) Endovascular intervention [6,7,8,9,10,11,12,15,16,17,19,20,21]. (**B**) Endarterectomy [6,21,22,23,25,28,29].

Other perioperative complications occurred in 13 studies [6,7,8,9,10,11,12,15,16,17,19,20,21] in the ET group and 7 studies [6,21,22,23,25,28,29] in the FAE group. A total of 3.85% (95% CI [1.70%, 8.48%]) (Figure 15A) patients in the ET group and 15.78% (95% CI [9.16%, 25.83%]) (Figure 15B) in the FAE experienced other perioperative complications. The cumulative odds ratio was 0.21 (95% CI [0.16–0.29], *p* < 0.05), in favour of ET.

**Figure 16:**
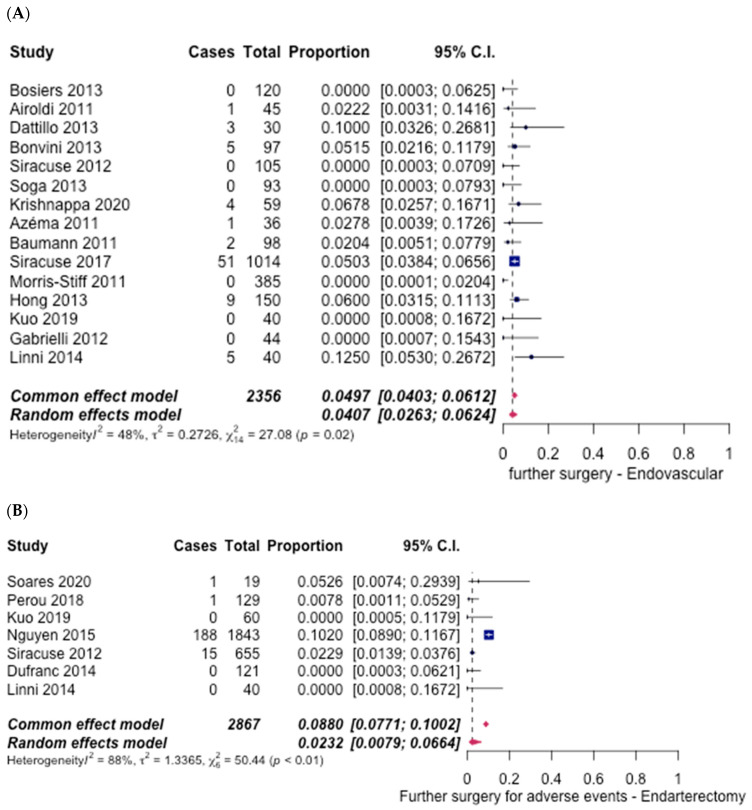
Forest plot for further surgery for adverse events. (**A**) Endovascular intervention [6,7,8,9,10,11,12,15,16,17,18,19,20,21]. (**B**) Endarterectomy [6,21,22,23,24,25,29].

Fifteen studies [6,7,8,9,10,11,12,15,16,17,18,19,20,21] mentioned the need for further surgery in the ET group, and an overall rate of 4.07% (95% CI [2.63%, 6.24%]) (Figure 16A) was noted. Similarly, only seven studies [6,21,22,23,24,25,29] reported the need for further surgery after FAE, with a total rate of only 2.32% (95% CI [0.79%, 6.64%]) (Figure 16B). The difference between the two groups was significant, in favour of FAE, with an odds ratio of 1.79 (95% CI [1.12–2.85], *p* < 0.05). 

## 4. Discussion

Our systematic review of ET of FAD versus endarterectomy had two main goals: technical success and safety. The results of this study show that although ET has high rates of technical success, it is still considered inferior in comparison to FAE. However, in terms of safety, both procedures are considered to be safe, with low rates of mortality and morbidity, but with lower rates of wound infection with ET.

Evidence suggests that the technical success of a procedure depends on the need for conversion to surgery or the need for revascularisation. As seen, the outcomes of technical success in both procedures were quite similar. The rate of revascularisation in FAE is considered nil compared with ET. This was highlighted in a study conducted by Kuo et al. [21]. Technical success is defined as residual stenosis of <30% on perioperative angiography, and loss of primary patency is defined as >50% restenosis. Contrary to the hypothesised association, this study showed a high technical success rate of endarterectomy of 95% to 100%. The results also confirm the claims of Linni et al. [6], whose study showed a technical success rate of 100% and significant postprocedural hemodynamic improvement in FAE. Although Kuo et al. 2018 [21] claimed FAE to be ‘the gold standard for femoral artery occlusion’, the paper also reported patients having essential blood loss requiring blood transfusion during the procedure (76.7%) and prolonged hospital stays. This did not change the favourability of FAE to ET, as the technical success and primary patency at two years were 94% and 57%, respectively. Symptom improvement has been defined differently in many studies reviewed but often implicates the ABPI (Ankle–Brachial Pressure index) improvement. The ABPI is a non-invasive method of measuring peripheral artery perfusion. It is the ratio of the blood pressure of the brachial artery (upper arm) and the posterior tibial and dorsalis pedis arteries (lower limb). In line with the results from Linni et al. [6], both femoral artery stenting and FAE improved the hemodynamic stability of the artery after the intervention, but FAE was more favourable in terms of technical success, postoperative hospital stays, and primary patency at 1 year. Technical success rates were 97.5% and 100% for the stenting and FAE groups, respectively. The 1-year primary patency rate of stented patients fell from 92.5% (at 30 days) to 80%, whereas FAE patients rested at 100% on both occasions. Ballotta et al. [3] reported a 7-year primary patency rate of 96% for FAE. Many studies also showed similar short-term and long-term patency rates, such as Kang et al. [31], who reported a 100% patency rate in 58 patients undergoing FAE at the 1-year and 5-year time points. These results provide a significant perspective on FAE intervention.

ET has also gained increasing attention as researchers aim at resolving the complications that involve restenosis of the stent. Recent literature has suggested that two-year secondary revascularisation rates are 73.2% [17] in patients with chronic limb-threatening ischemia. This was supported by Nisibe et al. [26], wherein an improvement in the mean ABPI was observed postoperatively in ET patients. The primary patency rate reported in the study at 24 months was 94% in ET patients, which aligns with our finding of ET being a vital alternative to FAE. 

It is common for patients undergoing ET to be started on blood-thinning medication, such as life-long aspirin (75–300 mg/dL) or Clopidogrel (75 mg/dL) in the event of thrombosis [32]. A study by Bosiers et al., 2013 [8], showed that patients on warfarin were at a 17.6% increased risk of acquiring wound site hematoma after endovascular intervention versus 0.9% of patients who were not on anti-coagulation medications. This finding is in line with an increase in the number of patients acquiring wound hematoma after ET compared with the number of patients with wound hematoma after FAE. New insight is required to determine which anti-coagulant is suitable for patients before ET to negate its side effects. Another important consideration is the duration of the hospital stay after the procedure. Studies have shown that the mean length of hospital stay for endovascular patients was three days and was prolonged if revascularisation was needed. One study in particular by Sajid et al. [33] demonstrated that patients undergoing endarterectomy required a mean of 8.4 days of hospital stay because of wound-related complications. Their postoperative complication rate was 24%, chiefly due to wound dehiscence and wound infection, requiring prolonged antibiotic therapy. Concerning complications of ET, a study conducted by Bosiers et al. [8] provided insight into stent fracture and its complications. It was noticed that there were about 4.2% stent fractures in the first year. No relationship was found between the occurrence of stent fracture and the type of stent used. Sustained clinical improvement was accepted at 12 months even though in-stent restenosis and stent fracture were evident. Most of these patients were not critical, and thus re-intervention was not performed.

ET is now recognised as an emerging treatment of femoral artery occlusions despite some limitations. High rates of restenosis remain the overpowering disadvantage of endovascular intervention in SFA occlusions [34]. Unlike coronary circulation, lower limb arteries involve long segments and multiple areas with a limited flow rate, predisposing the patient to restenosis. Thus, recent research has focused on devising tactics that can aid in the recanalisation of occluded superficial femoral arteries. Re-entry into true lumen risks damaging collateral blood vessels, thus creating a technical challenge [34]. 

Our review indicates a substantial advantage of safety for ET. A similar review conducted by Changal K et al. [35] offered a similar conclusion. However, ET and FAE were compared with a slightly different approach. ET was divided into routine or selective stenting. In their analysis, the rate of local complications was higher for FAE (22%) compared with both the endovascular treatments, which were 5% and 7%, respectively. The primary patency and target lesion revascularisation were comparable at maximum follow-up, with ET at 83.8% and FAE at 88%. Mortality was higher for FAE (23.1%) than for ET (5.3%). These findings support that ET may be better than FAE for FAD [35].

Evidence gathered by Malgor et al. [13] suggested that FAE is safer and more durable. In the study, it was noted that 4 out of the 262 patients developed acute MI but had successful coronary revascularisation [13]. Freedom from restenosis was outstanding at 92–93%. However, FAE alone did not provide a good limb salvage, and profundoplasty was needed to save patients from below-the-knee amputations. An RCT performed by Gabrielli R et al. [7] claimed the benefits of remote endarterectomy (RE) over ET of TransAtlantic Inter-Society Consensus for the Management of Peripheral Arterial Disease (TASC-II D) lesions in the femoropopliteal arteries. The primary patency rate of SFA treated with RE was revealed to be 76.5% (39 out of 51) versus only 56.8% (26 out of 44) in the ET group at 24 months. In the ET group, 13 re-occlusions were reported, compared with 11 in RE; thus, RE had a marginal advantage over ET [7]. Even though this was the case, they demonstrated that both endovascular and RE were safe and durable, and no major complications, such as limb amputation, were reported in either category. However, there was a significant difference in hospital length of stay. Most patients in the ET group were discharged the day after recovery, whereas patients who underwent FAE required at least two nights of recovery [7].

Medical interventions are evolving at an increased pace. Improvements in endovascular interventions are studied to reduce the incidence of stent fracture and in-stent restenosis. Topfer et al. [36] discussed the advancing technology of lithoplasty, in which balloon angioplasty is combined with sound waves to remove the atherosclerotic plaque. In the DISRUPT PAD Study [37], 94 patients had lithoplasty, and the target patency rate was 91.6%. Freedom from restenosis was 96.8%. 

Various other modes of treatment are being investigated, such as the use of high-speed rotational atherectomy for calcified lesions in the SFA [38]. Fukuda et al. [38] conducted a study to analyse the use of this technique in the treatment of a patient with heavily calcified lesions distal to a bypass graft on the SFA. The study mentioned the use of a Rotablator. A Rotablator is a newly designed atherectomy device that can help create space in the artery by removing the clot to facilitate the insertion of a stent if needed. However, there is a possibility of vessel perforation and distal embolisation. These complications usually arise when the burr size is larger with frequent passing and high-speed crossing. This evidence builds on the fact that future endarterectomies lean on using rotational endarterectomies combined with stenting, which provides an effective and acceptable result. The above-mentioned studies are ongoing and need more research to prove its advantage.

A limitation of this review is that the studies included in this systematic review predominantly included males. This was observed in a study conducted by Wang et al. [39], which included 11 males and 1 female for ET and FAE [39]. A study conducted by Hiramoto et al. [40] analysed the gender-specific risk factors for PAD. Their research suggested that amongst the general population, females had a higher risk for developing PAD. Although the reasons for this are unknown, it was established that one key marker was significantly elevated in females compared with males. This was the inflammatory marker CRP. It was also demonstrated that higher levels of CRP are linked to an increased risk of atherosclerotic outcomes. This also results in higher rates of functional decline, leading to poorer outcomes post treatment [40]. Moreover, another study that looked into the disparity in outcomes following ET between sexes postulated similar notions. A study by Choi et al. [41] stated that women experience higher rates of procedural complications, such as bleeding, wound infection, and vascular rupture. Adverse events in long-term follow up were also considered to be worse in the female population [41]. However, socio-cultural disparities that exist need to be considered for this observed disparity. In this study, it was noted that women presented with more advanced disease with more complex lesions. The aetiology for the observed trend may be unclear, but the researchers suggested that differences in anatomy and physiology, such as smaller vessel size; smaller calf muscles; a higher prevalence of asymptomatic disease; and increased rates of other comorbidities, such as arthritis and osteoporosis, may be a causative factor [41]. Therefore, the effects FAE and ET have on females cannot be generalised from the included studies. Furthermore, most studies include people between the ages of 60–70; likewise, it is not possible to generalise the findings to people outside this age group. This relates to the fact that people outside this age group have differences in anatomy, physiology, and prevalence of other comorbidities. Further studies should be conducted that allow researchers to extrapolate the results to the entire population. The follow-up period for the studies used in this paper had no uniformity, and some studies did not have sufficient follow-up periods. Certain studies have also reported short- or long-term mortality for FAE and ET treatments; however, it is not known whether mortality occurred because of the surgery or other complications. Future research can focus on examining the causes of death in these patients. 

Another factor that plays a key role in determining the uptake of a certain procedure is its financial implications. Tang et al. [42] performed a multicentre retrospective cost analysis of ET compared with open surgery. The research suggested that there was a significant difference in the costs associated with the procedures. ET was considered to be less expensive. The bulk of the costs associated with ET included the use of prosthetics and stents. However, an increased need for experienced clinical staff (medical and nursing), critical care utilisation, and operating theatre costs in open surgeries outweigh the costs incurred in ET [42]. 

Within our study, there exists a certain level of selection and confirmation bias. Since all studies were selected manually with a pre-existing hypothesis, there may be a possibility of selecting only those favouring the hypothesis. This was, however, minimised by conducting a comprehensive literature search across the different databases. Because of the lack of meta-analyses performed in the study, the heterogeneity amongst the studies included is not considered in the data summaries.

## 5. Conclusions

Substantial evidence on the efficacy and safety of ET cannot be established from this study. Because of insufficient evidence on long-term outcomes, a fair comparison cannot be made. This study does not prove that ET is superior to FAE. Moreover, a detailed analysis of the financial implications and the learning curve associated with ET is required to draw well-supported conclusions as to which technique is superior. 

## Figures and Tables

**Table 1:** Summary of studies included and patient characteristics.

	Endovascular Intervention	Endarterectomy
No. of Studies Selected	n = 14	n = 12
No. of RCT Studies Selected	n = 1	n = 1
No. of Retrospective Studies Selected	n = 10	n = 11
No. of Prospective Studies Selected	n = 3	n = 0
No. of Patients	n = 2496	n = 4630
No. of Males	n = 1763	n = 3098
No. of Females	n = 978	n = 1711
Mean Age of Patients	68.24	69.16

## Data Availability

Not applicable.

## Supplementary Materials

The following are available online at https://www.mdpi.com/article/10.3390/hematolrep14020026/s1, Table S1: Summary of quality assessment of the RCTs. Table S2: Summary of quality assessment of Case series. File S1: Search Strategy used for the different databases. File S2: Newcastle-Ottawa Quality Assessment Scale: Case Control Studies. File S3: Quality assessment of RCTs.
