# Supplementary material for: Comparative Analysis of Endovascular Intervention and Endarterectomy in Patients with Femoral Artery Disease: A Systematic Review and Meta-Analysis
Source: Hematol Rep. 2022 Jun 1;14(2):179–202. doi: 10.3390/hematolrep14020026 (PMC9222618; doi:10.3390/hematolrep14020026)
Supplement: Supplementary file 1 [file hematolrep-14-00026-s001.zip › hematolrep-1642636-supplementary/File S1.pdf]

File S1: Search Strategy used for the different databases.

PubMed Search Strategy

"endarterectomy*"[MeSH] OR "endovascular procedure*"[MeSH] OR "stent*"[MeSH] OR "endarterectomy*"[tw] OR "endovascular"[tw] OR "stent*"[tw] AND "femoral artery*"[MeSH] OR "peripheral arterial disease*"[MeSH] OR "femoral artery*"[tw]

Filters: 2011-2021

6192 results

Scopus Search Strategy

endovascular AND versus AND endarterectomy AND for AND femoral AND artery AND disease

1607 results

Embase Search Strategy

("common femoral artery*" and ("endovascular*" or "endarterectomy*")).mp. [mp=title, abstract, heading word, drug trade name, original title, device manufacturer, drug manufacturer, device trade name, keyword, floating subheading word, candidate term word]

740 results
