# Supplementary material for: Comparative Analysis of Endovascular Intervention and Endarterectomy in Patients with Femoral Artery Disease: A Systematic Review and Meta-Analysis
Source: Hematol Rep. 2022 Jun 1;14(2):179–202. doi: 10.3390/hematolrep14020026 (PMC9222618; doi:10.3390/hematolrep14020026)
Supplement: Supplementary file 1 [file hematolrep-14-00026-s001.zip › hematolrep-1642636-supplementary/File S3.pdf]

File S3: Quality assessment of RCTs

**Criteria**	**Yes**	**No**	**Unclear**
1. Was the assignment to the treatment groups really random?			
2. Was the treatment allocation concealed?			
3. Were the groups similar at baseline in terms of prognostic factors?			
4. Were the eligibility criteria specified?			
5. Was the intervention (and comparison) clearly defined?			
6. Were the groups treated in the same way apart from the intervention received?			
7. Was there a follow-up period > 30 days?			
8. Was the outcome assessor blinded to the treatment allocation?			
9. Was the care provider blinded?			
10. Were the patients blinded?			
11. Were the point estimates and measures of variability presented for the primary outcome measures?			
12. Was the withdrawal/drop-out rate likely to cause bias?			
13. Did the analyses include an intention-to-treat analysis?			
14. Was the operation undertaken by somebody experienced in performing the procedure?			
