# Supplementary material for: Comparative Analysis of Endovascular Intervention and Endarterectomy in Patients with Femoral Artery Disease: A Systematic Review and Meta-Analysis
Source: Hematol Rep. 2022 Jun 1;14(2):179–202. doi: 10.3390/hematolrep14020026 (PMC9222618; doi:10.3390/hematolrep14020026)
Supplement: Supplementary file 1 [file hematolrep-14-00026-s001.zip › hematolrep-1642636-supplementary/Table S1.pdf]

Table S1: Summary of quality assessment of the RCTs

Criteria	Yes	No	Unclear
1. Was the assignment to the treatment groups really random?	2[6,7]		
2. Was the treatment allocation concealed?	1[6]		1[7]
3. Were the groups similar at baseline in terms of prognostic factors?	2[6,7]		
4. Were the eligibility criteria specified?	2[6,7]		
5. Was the intervention (and comparison) clearly defined?	2[6,7]		
6. Were the groups treated in the same way apart from the intervention received?	2[6,7]		
7. Was there a follow-up period > 30 days?	2[6,7]		
8. Was the outcome assessor blinded to the treatment allocation?		1[6]	1[7]
9. Was the care provider blinded?		1[6]	1[7]
10. Were the patients blinded?		1[6]	1[7]
11. Were the point estimates and measures of variability presented for the primary outcome measures?	2[6,7]		
12. Was the withdrawal/drop-out rate likely to cause bias?	1[6]		1[7]
13. Did the analyses include an intention-to-treat analysis?	1[6]		1[7]
14. Was the operation undertaken by somebody experienced in performing the procedure?	2[6,7]		
