# Supplementary material for: Comparative Analysis of Endovascular Intervention and Endarterectomy in Patients with Femoral Artery Disease: A Systematic Review and Meta-Analysis
Source: Hematol Rep. 2022 Jun 1;14(2):179–202. doi: 10.3390/hematolrep14020026 (PMC9222618; doi:10.3390/hematolrep14020026)
Supplement: Supplementary file 1 [file hematolrep-14-00026-s001.zip › hematolrep-1642636-supplementary/Table S2.pdf]

Table S2: Summary of quality assessment of Case series

Selection

Study	First Author	Is the case definition adequate	Representativeness of the cases	Selection of controls	Definition of controls
A single-center experience in the eversion femoral endarterectomy	Tony R Soares	*	*	*	*
4-French-compatible endovascular material is safe and effective in the treatment of femoropopliteal occlusive disease: results of the 4-EVER trial	Marc Bosiers	*	*		
A novel device for true lumen re-entry after subintimal recanalization of superficial femoral arteries: first-in-man experience and technical description	Flavio Airoldi	*	*	*	*
Acute and medium-term outcomes of endovascular therapy of obstructive disease of diverse etiology of the common femoral artery	Philip B. Dattilo	*	*	*	*
Angioplasty and provisional stent treatment of common femoral artery lesions	Robert F. Bonvini	*	*	*	*
Assessing the perioperative safety of common femoral endarterectomy in the endovascular era	Jeffrey J. Siracuse	*	*	*	*

Clinical outcome after endovascular treatment for isolated common femoral and popliteal artery disease	Yoshimitsu Soga	*	*	*	*
Common femoral artery endarterectomy for lower-extremity ischemia: evaluating the need for additional distal limb revascularization	Malgor RD	*	*	*	*
Efficacy and safety of adjunctive drug-coated balloon therapy in endovascular treatment of common femoral artery disease	Hafiz M Imran	*	*		*
Endovascular Interventions to Superficial Femoral Artery Occlusion: Different Approaches, Technique, and Follow-up	Santhosh Krishnappa	*	*	*	*
Endovascular repair of common femoral artery and concomitant arterial lesions	Azéma L	*	*	*	*
Endovascular treatment of common femoral artery obstructions	Frederic Baumann		*		*
Endovascular treatment of the common femoral artery in the Vascular Quality Initiative	Jeffrey J. Siracause	*	*	*	*
Femoral Bifurcation Endarterectomy with Transection-Eversion of the Superficial Femoral Artery: Technique and Results	Perou S	*	*	*	*
Long-term clinical outcome following lower limb arterial angioplasty	Morris-Stiff G	*	*	*	*
Midterm outcomes of subintimal angioplasty supported by primary proximal stenting for chronic total occlusion of the superficial femoral artery	Hong SJ	*	*	*	*

Outcome of drug-eluting balloon angioplasty versus endarterectomy in common femoral artery occlusive disease	Kuo TT	*	*	*	*
Postoperative complications after common femoral endarterectomy	Nguyen BN	*	*	*	*
Results for primary bypass versus primary angioplasty/stent for intermittent claudication due to superficial femoral artery occlusive disease	Siracuse JJ	*	*	*	*
Results of common femoral artery thromboendarterectomy evaluation of a traditional surgical management in the endovascular era	Wieker CM	*	*	*	*
Technique and results of femoral bifurcation endarterectomy by eversion	Dufranc J.	*	*		
The Advantage of Common Femoral Endarterectomy Alone or Combined with Endovascular Treatment	Jun Hyung Kim	*	*	*	*
The Role of Common Femoral Artery Endarterectomy in the Endovascular Era	Toshiya Nishibe	*	*	*	*
Two-Year Outcomes of Endovascular Interventions of the Common Femoral Artery: A Retrospective Analysis From Two Medical Centers	Nicolas W Shammas	*	*	*	*

Comparability

Study	First Author	Comparability of cases and control on the basis of the design or analysis
A single-center experience in the eversion femoral endarterectomy	Tony R Soares	**
4-French-compatible endovascular material is safe and effective in the treatment of femoropopliteal occlusive disease: results of the 4-EVER trial	Marc Bosiers	
A novel device for true lumen re-entry after subintimal recanalization of superficial femoral arteries: first-in-man experience and technical description	Flavio Airoldi	**
Acute and medium-term outcomes of endovascular therapy of obstructive disease of diverse etiology of the common femoral artery	Philip B. Dattilo	**
Angioplasty and provisional stent treatment of common femoral artery lesions	Robert F. Bonvini	**
Assessing the perioperative safety of common femoral endarterectomy in the endovascular era	Jeffrey J. Siracuse	**
Clinical outcome after endovascular treatment for isolated common femoral and popliteal artery disease	Yoshimitsu Soga	**
Common femoral artery endarterectomy for lower-extremity ischemia: evaluating the need for additional distal limb revascularization	Malgor RD	**

Efficacy and safety of adjunctive drug-coated balloon therapy in endovascular treatment of common femoral artery disease	Hafiz M Imran	**
Endovascular Interventions to Superficial Femoral Artery Occlusion: Different Approaches, Technique, and Follow-up	Santhosh Krishnappa	**
Endovascular repair of common femoral artery and concomitant arterial lesions	Azéma L	**
Endovascular treatment of common femoral artery obstructions	Frederic Baumann	
Endovascular treatment of the common femoral artery in the Vascular Quality Initiative	Jeffrey J. Siracause	**
Femoral Bifurcation Endarterectomy with Transection-Eversion of the Superficial Femoral Artery: Technique and Results	Perou S	**
Long-term clinical outcome following lower limb arterial angioplasty	Morris-Stiff G	**
Midterm outcomes of subintimal angioplasty supported by primary proximal stenting for chronic total occlusion of the superficial femoral artery	Hong SJ	**
Outcome of drug-eluting balloon angioplasty versus endarterectomy in common femoral artery occlusive disease	Kuo TT	**
Postoperative complications after common femoral endarterectomy	Nguyen BN	**
Results for primary bypass versus primary angioplasty/stent for intermittent claudication due to superficial femoral artery occlusive disease	Siracuse JJ	**
Results of common femoral artery thromboendarterectomy evaluation of a traditional surgical management in the endovascular era	Wieker CM	**

Technique and results of femoral bifurcation endarterectomy by eversion	Dufranc J.	**
The Advantage of Common Femoral Endarterectomy Alone or Combined with Endovascular Treatment	Jun Hyung Kim	**
The Role of Common Femoral Artery Endarterectomy in the Endovascular Era	Toshiya Nishibe	**
Two-Year Outcomes of Endovascular Interventions of the Common Femoral Artery: A Retrospective Analysis From Two Medical Centers	Nicolas W Shammas	**

Outcome

Study	First Author	Assessment of outcome	was follow up long enough for outcomes to occur	Adequacy of follow up fo cohorts
A single-center experience in the eversion femoral endarterectomy	Tony R Soares	*	*	*
4-French-compatible endovascular material is safe and effective in the treatment of femoropopliteal occlusive disease: results of the 4-EVER trial	Marc Bosiers	*	*	*
A novel device for true lumen re-entry after subintimal recanalization of superficial femoral arteries: first-in-man experience and technical description	Flavio Airoldi	*		
Acute and medium-term outcomes of endovascular therapy of obstructive disease of diverse etiology of the common femoral artery	Philip B. Dattilo	*	*	*
Angioplasty and provisional stent treatment of common femoral artery lesions	Robert F. Bonvini	*	*	*

Assessing the perioperative safety of common femoral endarterectomy in the endovascular era	Jeffrey J. Siracuse	*	*	*
Clinical outcome after endovascular treatment for isolated common femoral and popliteal artery disease	Yoshimitsu Soga	*	*	*
Common femoral artery endarterectomy for lower-extremity ischemia: evaluating the need for additional distal limb revascularization	Malgor RD	*	*	*
Efficacy and safety of adjunctive drug-coated balloon therapy in endovascular treatment of common femoral artery disease	Hafiz M Imran	*	*	
Endovascular Interventions to Superficial Femoral Artery Occlusion: Different Approaches, Technique, and Follow-up	Santhosh Krishnappa	*	*	*
Endovascular repair of common femoral artery and concomitant arterial lesions	Azéma L	*	*	*
Endovascular treatment of common femoral artery obstructions	Frederic Baumann	*		
Endovascular treatment of the common femoral artery in the Vascular Quality Initiative	Jeffrey J. Siracause	*	*	
Femoral Bifurcation Endarterectomy with Transection-Eversion of the Superficial Femoral Artery: Technique and Results	Perou S	*	*	*
Long-term clinical outcome following lower limb arterial angioplasty	Morris-Stiff G	*		*
Midterm outcomes of subintimal angioplasty supported by primary proximal stenting for chronic total occlusion of the superficial femoral artery	Hong SJ	*	*	*

Outcome of drug-eluting balloon angioplasty versus endarterectomy in common femoral artery occlusive disease	Kuo TT	*	*	*
Postoperative complications after common femoral endarterectomy	Nguyen BN	*	*	*
Results for primary bypass versus primary angioplasty/stent for intermittent claudication due to superficial femoral artery occlusive disease	Siracuse JJ	*	*	*
Results of common femoral artery thromboendarterectomy evaluation of a traditional surgical management in the endovascular era	Wieker CM	*	*	*
Technique and results of femoral bifurcation endarterectomy by eversion	Dufranc J.	*	*	
The Advantage of Common Femoral Endarterectomy Alone or Combined with Endovascular Treatment	Jun Hyung Kim	*	*	*
The Role of Common Femoral Artery Endarterectomy in the Endovascular Era	Toshiya Nishibe	*	*	
Two-Year Outcomes of Endovascular Interventions of the Common Femoral Artery: A Retrospective Analysis From Two Medical Centers	Nicolas W Shammas	*	*	*
